# Terahertz Wave Propagation in a Nanotube Conveying Fluid Taking into Account Surface Effect

**DOI:** 10.3390/ma6062393

**Published:** 2013-06-10

**Authors:** Ye-Wei Zhang, Tian-Zhi Yang, Jian Zang, Bo Fang

**Affiliations:** 1 Department of Astronautics, Shenyang Aerospace University, Shenyang 110136, China; E-Mails: zhangyewei1218@126.com (Y.-W.Z.); yangtz@sau.edu.cn (T.-Z.Y.); zangjian106@163.com (J.Z.); 2 School of Astronautics, Harbin Institute of Technology, Harbin 100124, China

**Keywords:** single-walled carbon nanotube, surface effect, nonlocal Timoshenko beam theory, terahertz wave

## Abstract

In nanoscale structure sizes, the surface-to-bulk energy ratio is high and the surface effects must be taken into account. Surface effect plays a key role in accurately predicting the vibration behavior of nanostructures. In this paper, the wave behaviors of a single-walled carbon nanotube (CNT) conveying fluid are studied. The nonlocal Timoshenko beam theory is used and the surface effect is taken into account. It is found that the fluid can flow at a very high flow velocity and the wave propagates in the terahertz frequency range. The surface effects can significantly enhance the propagating frequency. This finding is different from the classical model where the surface effect is neglected.

## 1. Introduction

The carbon nanotube is a key structure and has many potential applications. Very recently, hollow geometry has been considered regarding many new applications in biological devices such as nanofluid conveyance and drug delivery [1,2]. The behavior of the ﬂuid inside carbon nanotubes (CNTs) is expected to be significantly different from the behavior of the ﬂuid in macro pipe at low velocities because of the very small diameter of CNTs, and therefore it is a challenging research topic in a nano system. Many studies of the interaction between fluid and nanotube have been investigated by using classical and nonlinear elastic theory [3,4,5,6,7,8,9,10].

However, as the structure sizes are in nano scale, the surface-to-bulk energy ratio is high and the surface effects must be taken into account. Therefore, surface effects on the performance of nanostructures have been studied widely. For example, Lee and Chang [11] used nonlocal Timoshenko beam theory to study the vibration behavior of nanotubes with surface effect. They observed that the frequency ratio increases when the surface effect is taken into account. Wang [12] formulated the dynamics of fluid-conveying nanotubes. They found that the natural frequencies increase due to the the presence of surface effect. Narendar *et al.* [13,14] compared the nonlocal wave properties of nanotubes and nanoplate with and without surface effect. They found that the flexural wavenumbers with surface effect become higher. Therefore, it is believed that surface effect plays a key role in accurately predicting the vibration behavior of nanostructures. However, the discussion of surface effect on the dynamics of nanotube conveying fluid was rather limited. In this paper, the combined effects of surface property and fluid-CNT interaction on wave propagation are analyzed in detail, which is a different approach from the previous models [15,16,17,18].

On the other hand, very recently, it has been found that the fluid can flow at a very high flow velocity and the wave propagates in the terahertz frequency range [19]. Thus, the shear deformation and rotary inertia will significantly influence the high wave propagating frequency. This suggests that the nonlocal timoshenko beam is more accurate for analyzing the dynamical behavior of a nanotube.

## 2. Timoshenko Model for Nanotube Conveying Fluid

**Figure 1:**
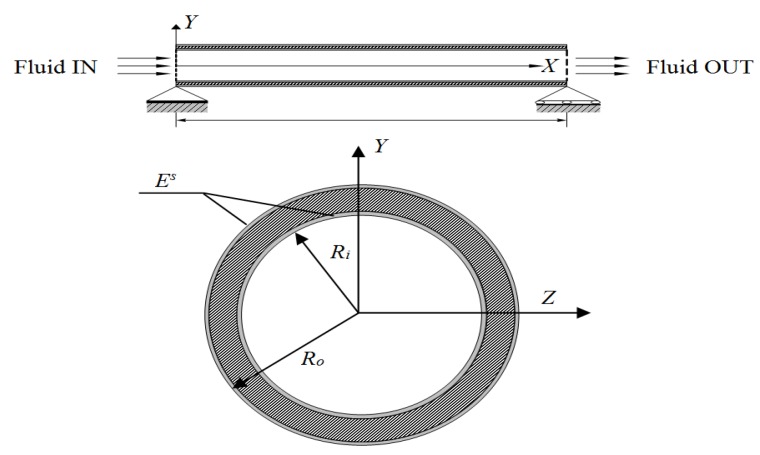
Schematic diagram of a nanotube conveying fluid.

A schematic diagram of a nanotube conveying fluid is shown in Figure 1. The nanotube has the length L. *M_c_* and *M_F_* are the mass per unit axial length. In this paper, the dynamic behavior of the nanotube is analyzed by using the nonlocal Timoshenko beam theory

(1) $$EI^{*}\frac{\partial^{2}\psi }{\partial x^{2}}+\left[1-{\left(e_{0}a\right)}^{2}\frac{\partial^{2}}{\partial x^{2}}\right]\left[KGA\left(\frac{\partial Y}{\partial x}-\psi \right)-J_{C}\frac{\partial^{2}\psi }{\partial t^{2}}\right]-J_{F}\frac{\partial^{2}\psi }{\partial t^{2}}=0$$

(2) $$\frac{\partial }{\partial x}KGA\left(\frac{\partial Y}{\partial x}-\psi \right)+H\frac{\partial^{2}Y}{\partial x^{2}}=M_{C}\frac{\partial^{2}Y}{\partial t^{2}}+M_{F}U_{F}^{2}\frac{\partial^{2}Y}{\partial x^{2}}+2M_{F}U_{F}\frac{\partial^{2}Y}{\partial x\partial t}+M_{F}\frac{\partial^{2}Y}{\partial t^{2}}-M_{C}{\left(e_{0}a\right)}^{2}\frac{\partial^{4}Y}{\partial t^{2}\partial x^{2}}$$

in which *ψ* is the rotation angle of the cross section perpendicular to the longitudinal axis and *Y* is the transverse displacement. *J*_C_ and *J*_F_ are the mass moment of inertia for the nanotube conveying fluid. *e*_0_ is the constant appropriate to each material and a the internal characteristic length. The value of *e*_0_ can be identified by experiments. If *e*_0_*a* = 0, the Equations (1) and (2) reduce to the classical Timoshenko beam. *U*_F_ is the flow velocity of the fluid inside. G is the shear modulus, A is the cross section area of the CNT and K is the shear correction factor. EI^*^ is the flexural rigidity induced by the surface energy in nanotubes, which can be expressed as [12]

(3) $$EI^{*}=EI+\pi E^{S}(R_{i}^{3}+R_{o}^{3})$$

where *EI* is deﬁned as the ﬂexural rigidity of the empty nanotube without surface layers. *E^s^* is the surface elastic modulus, *Ro* and *Ri* are the outer and inner radius of the nanotube, respectively. *H* is the surface parameter and defined by

(4) $$H=4\tau (R_{i}+R_{o})$$

in which *τ* is determined by the residual surface tension.

For analyzing the ultrasonic wave dispersion characteristics in nanotube, the wave solution for the displacement can be assumed as

(5) $$Y(x,t)=\tilde{y}e^{-j(kx-\omega t)}$$

(6) $$\psi (x,t)=\tilde{\psi }e^{-j(kx-\omega t)}$$

in which $\tilde{y}$ and $\tilde{\psi }$ are the amplitude of the wave motion, *k* is the wavenumber and *ω* is the angular frequency of the wave motion. *j* denotes the imaginary symbol $\sqrt{–1}$ Substituting Equations (5) and (6) into (1) and (2) yield

(7) $$\left[\begin{array}{cc} Q_{11} & Q_{12}\\ Q_{21} & Q_{22} \end{array}\right]\left\{\begin{array}{c} \tilde{y}\\ \tilde{\psi } \end{array}\right\}=\left\{\begin{array}{c} 0\\ 0 \end{array}\right\}$$

in which

$$\begin{array}{l} Q_{11}=KGAk^{2}+Hk^{2}-M_{c}\omega^{2}-M_{F}{U_{F}}^{2}k^{2}+2M_{F}U_{F}k\omega -M_{F}\omega^{2}-M_{c}{\left(e_{0}a\right)}^{2}k^{2}\omega^{2};\\ Q_{12}=-KGAkj;\\ Q_{21}=-KGAkj-{\left(e_{0}a\right)}^{2}KGAk^{3}j;\\ Q_{22}=-EI^{*}k^{2}-KGA+J_{c}\omega^{2}+J_{F}\omega^{2}+{\left(e_{0}a\right)}^{2}J_{c}k^{2}\omega^{2}-{\left(e_{0}a\right)}^{2}KGAk^{2}; \end{array}$$

The resultant Equation (7) also can be re-written as an algebra equation of wavenumber *k*

(8) $$[S_{3}]k^{3}+[S_{2}]k^{2}+[S_{1}]k+[S_{0}]=0$$

where

$$\begin{array}{l} \left[S_{3}\right]=\left[\begin{array}{cc} 0 & 0\\ -{\left(e_{0}a\right)}^{2}KGAj & 0 \end{array}\right];\\ \left[S_{2}\right]=\left[\begin{array}{cc} KGA-M_{c}{\left(e_{0}a\right)}^{2}\omega^{2}-M_{F}U_{F}{}^{2}+H & 0\\ 0 & -EI^{*}+{\left(e_{0}a\right)}^{2}J_{c}\omega^{2}-{\left(e_{0}a\right)}^{2}KGA \end{array}\right];\\ \left[S_{1}\right]=\left[\begin{array}{cc} 2M_{F}U_{F}\omega & -KGAj\\ KGAj & 0 \end{array}\right];\\ \left[S_{0}\right]=\left[\begin{array}{cc} -M_{c}\omega^{2}-M_{F}\omega^{2} & 0\\ 0 & -KGA+J_{c}\omega^{2}+J_{F}\omega^{2} \end{array}\right]; \end{array}$$

The frequency at which the imaginary part of the wavenumber becomes real is called the shear cut-off frequency and can be obtained by setting *k* = 0, which yields $[S_{0}]=0$. Thus, the cut-off frequencies of flexural and shear modes can be obtained

(9) $$\omega_{c}^{flexural}=0,\omega_{c}^{shear}=\sqrt{\frac{GAK}{J_{C}+J_{F}}}$$

Equation (9) indicates that the nonlocal size effect *e*_0_*a* and the surface effect are independent on the cut-off frequencies.

## 3. Numerical Results and Discussion

In this section, the dynamics of the nanotube conveying fluid is numerically demonstrated. The parameters are assumed as *K* = 5/6; *G* = 27 GPa; *E* = 3.5 TPa; *ρ* = 2700 kg/m^3^; *ρ* = 2000 kg/m^3^; *τ* = 0.9108 N/m; *E*_s_ = 5.1882 N/m; *R*_0_ = 3.5 nm; *R_i_* = 3.4 nm; *U_f_* = 1000 m/s and *e*_0_*a* = 0.5 nm.

**Figure 2:**
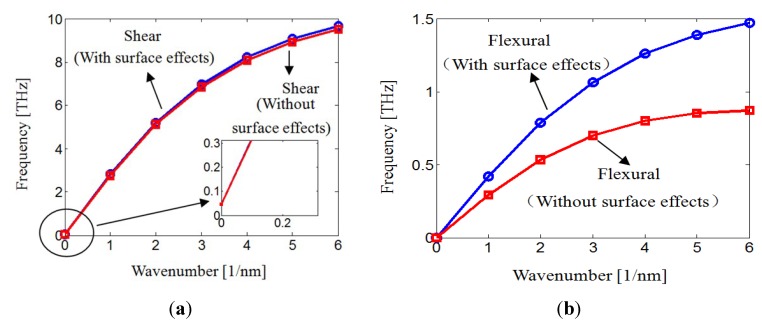
Wave dispersion of nanotube conveying fluid with and without surface effects.

The dispersion curves are calculated numerically. Figure 2a,b are the first fundamental shear and flexural modes, respectively. It is shown that both the shear mode and flexural modes all start from zero wavenumber, indicating that they propagate at the same time. The shear frequency and flexural frequency increase as the wavenumber increases. On the other hand, it is seen in Figure 2a that the flexural wave frequencies are slightly enhanced in the presence of surface effect.

In contrast, in Figure 2b, the surface effect has a significant influence on the flexural mode. The presence of the surface effects lead to drastically enhanced terahertz flexural wave frequencies. For example, for a given wavenumber 2 nm, the flexural wave frequency without surface effect is 0.53 THz. On the other hand, the enhanced flexural wave frequency is 0.79 THz by taking the surface into account. Thus, the as the structure sizes are in nanoscale, the surface effects cannot be neglected.

**Figure 3:**
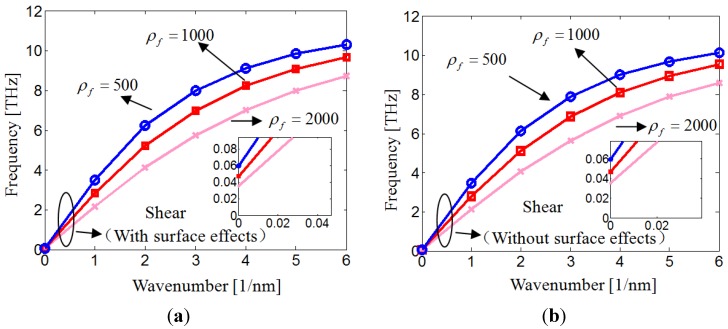
Shear wave dispersion of nanotube conveying fluid with different fluid densities.

Figure 3 depicts the effects of fluid density on the shear wave dispersion. It is seen in Figure 3 that the shear wave frequencies decrease as the fluid becomes denser for both of the shear wavemodes.

**Figure 4:**
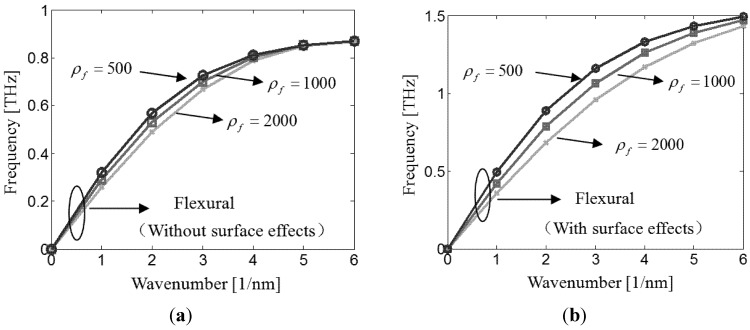
Flexural wave dispersion of nanotube conveying fluid with different fluid densities.

Figure 4a shows the variation of the flexural wave frequency with the wavenumber without surface effect. It is found that as the fluid become denser, the flexural wave frequency slightly decreases. On the other hand, it can be seen from Figure 4b that, in the presence of the surface effects, the flexural wave behavior is drastically altered. The denser fluid leads to a smaller flexural wave frequency.

## 4. Conclusions

In the present paper, the effect of surface effect on the teraherz wave propagation in fluid conveying nanotube is studied. The nonlocal Timoshenko beam model is adopted to derive the governing equation. The cut-off frequencies of flexural and shear modes is derived analytically. It was found that the nonlocal size effect *e*_0_*a* and surface effect are independent of the cut-off frequencies. Numerical results show that the presence of the surface effects yield enhanced terahertz flexural wave frequencies. The shear wave frequency slightly decreases as the fluid become denser for both the shear and flexural wavemodes. In the presence of the surface effects, the flexural wave frequency increases as the flow velocity increases.
